# The effects of anti-galactagogue (sage, parsley extract) and anti-inflammatory (echinacea extract) feed supplements on prolactin levels and fertility in the re-pregnancy of lactating Merino ewes

**DOI:** 10.1007/s11250-025-04308-8

**Published:** 2025-02-13

**Authors:** Metehan Kutlu, Neffel Kürşat Akbulut

**Affiliations:** Department of Obstetrics and Gynaecology, Faculty of Veterinary Medicine, Necmettin Erbakan University, Konya, Turkey

**Keywords:** Ewe, Fertility, Prolactin, Parsley, Sage

## Abstract

The purpose of this study was to examine the effects of anti-galactagogue (sage, parsley extract) and anti-inflammatory (echinacea extract) feed supplements on prolactin levels and fertility in the re-pregnancy of lactating Merino ewes. Eighty ewes were randomly assigned to one of two groups: control and treatment. In treatment group (Stop Lactin) group (n = 40) ewes were treated with Stop Lactin® 60 ml on day 0 orally by syringe. The control group (Control) (n = 40), ewes were not treated with any anti-galactagogue feed supplements. On day 0, a vaginal sponge containing 60 mg of medroxyprogesterone acetate was inserted for seven days. On day 7, all ewes received an intramuscular injection of 500 IU PMSG. The study results indicated no statistically significant differences between the Control group and Stop Lactin group in terms of conception rates (87.2% and 78.1%), late embryonic-early fetal mortality rate (20.6% and 8.0%), lambing rate (79.4% and 92%), twin rate (29.6% and 13.0%) and litter size (1.30 and 1.13). Estrus rates (97.5% and 82.1%, p = 0.050) tended to be higher and pregnancy rates (85.0% and 64.1%, p = 0.037) was found to be significant in the control group compared to the Stop Lactin group. The effect of treatment (P = 0.209) on serum prolactin levels and the interaction between treatment × day (P = 0.874) were both found to be insignificant. It is concluded that anti-galactagogue and anti-inflammatory feed supplements did not reduce prolactin concentration and did not improve fertility in lactating Merino ewes.

## Introduction

In order to enhance the efficiency of sheep production, it has been suggested to increase the lambing frequency in alignment with the ewe's 5-month gestation period (Goff et al. 2014). For this purpose, the two most commonly used accelerated lambing programs are “three lambings in two years” (Alaçam 1993) and five lambings in three years—Cornell STAR© systems (Smith 2006). If these programs are implemented, lambing intervals of approximately every 8 months or every 7.2 months are achievable, respectively. However, postpartum anoestrus in ruminants significantly impacts reproductive performance and results in economic losses for producers (Pérez-Hernández et al. 2002). The timing of lamb weaning is a critical factor that directly affects the duration of post-lambing anoestrus in ewes (Ronquillo et al. 2008). Similarly, suckling in ewes has been shown to prolong the postpartum anovulatory period (Pérez-Hernández et al. 2009). The main cause for this is the suppressive effect of the suckling stimulus (Griffith and Williams 1996; Williams 1990). Adrenocorticotropic hormone (ACTH), prolactin and endogenous opioid peptides, which are among the known inhibitors of the gonadotropin-releasing hormone/luteinizing hormone (GnRH/LH) system during lactation, play very important roles in reproduction (Ascari et al. 2016; Dobek et al. 2013; McNeilly 1980). It has been suggested that defining the primary inhibitor involved in modulating reproductive function is challenging (Dobek et al. 2013).

In ewes, lactation or suckling leads to elevated prolactin levels during the postpartum period, which may suppress gonadotropins and hinder pregnancy (Mandiki et al. 1990). In this context, one of the options that improve fertility is limited suckling. There are studies suggesting that limited suckling of lambs shortens the interval between the first postpartum estrus and ovulation (Mandiki et al. 1989; Morales-Terán et al. 2004; Schirar et al. 1989), there are also studies reporting that it has no effect (Arroyo-Ledezma et al. 2000).

Moreover, the systemic administration of prolactin-release inhibitors and their effects on mammary gland involution have been studied. These inhibitors are dopamine D2 receptor agonists, such as cabergoline, bromocriptine or quinagolide, which inhibit the release of prolactin from the pituitary gland. Consequently, this counteracts the galactopoietic effects of prolactin (Lacasse et al. 2012). It is noteworthy that daily oral administration of cabergoline did not significantly reduce prolactin levels or improve estrous response and pregnancy rates in ewes (Saat et al. 2024). Quinagolide has been demonstrated to reduce milk production (Lacasse et al. 2012; Ollier et al. 2013, 2014) and improve resistance to intra-mammary infection after dry-off (Ollier et al. 2015). Despite these promising results, the current treatment regimen of prolactin-relase inhibitors, which involves administration once or twice daily for several days, is not practical for field applications (Bertulat et al. 2017; Ollier et al. 2015).

Alternatively, plants such as sage leaves, peppermint oil, chasteberry, parsley and jasmine can be used to reduce milk production (Eglash 2014). Stop Lactin® (Raizup, Nutrinova, France) is a herbal mixture prepared based on the lactation suppressant properties of sage, the inhibition of parsley leaves on the prolactin hormone, and the positive effects of echinacea on somatic cell numbers and immunoglobulin in the serum. Sage (*Salvia officinalis*) leaf comprises tannins (salviatannin), essential oils (including alpha-thujone, beta-thujone, 1,8 cineole, and camphor), flavones, phenolic acids, phenylpropanoid glycosides, triterpenoids, and diterpenes (Drugs and Lactation Database (LactMed®) [Internet] 2006). Sage, which has a potential phytoestrogenic and antiperspirant effect, may provide benefits at the point of weaning (Yarnell 1997). Another herb that has an antigalactagogue effect is parsley. Parsley (*Petroselinum Crispum*) contains in ascorbic acid, carotenoids, flavonoids, apiole, terpenoic compounds, coumarin, phenylpropanoids, phthalides, tocopherol, and furanocoumarins (Akpaso et al. 2023). Oral capsules containing sage and parsley capsules are said to reduce milk flow in humans. However, there are no scientifically valid clinical studies (Brodribb 2018; Eglash 2014). Both parsley and sage, known as antigalactagogues, are used to prevent or reduce milk secretion. They are recommended for preventing postpartum mastitis and lessen engorgement (Mohanty et al. 2014). Additionally, it was revealed in a study conducted on rats that parsley has an estrogenic effect. This effect has the potential to increase fertility through its effect on estrogen hormone levels at high doses (Akpaso et al. 2023). Echinacea, a widely used plant with demonstrated antimicrobial effects, stimulates many immune cells, including macrophages and natural killer cells (Stanisavljević et al. 2009). The chemical components of echinacea species include phenolic compounds, flavonoids, essential oils, polyacetylenes, nitrogenous compounds and polysaccharides (Çalışkan and Odabaş 2011). In a rat study, echinacea was found to significantly elevate serum IgG levels (Yamada et al. 2011).

It has been thought that the use of parsley and sage for weaning ewes may prevent/reduce the negative effects of prolactin. The aim of the presented study is to reveal the effect of anti-galactagogue (sage, parsley extract) and anti-inflammatory (echinacea extract) as feed supplements on prolactin levels and fertility in the re-pregnancy of lactating Merino ewes.

## Material and methods

The present study was conducted with approval of Selçuk University Animal Experiments Local Ethics Committee, Konya, Türkiye (2024/012).

### Animals

This study was conducted in a commercial sheep farm (Lat: 37° 86′ 44.06» N, Long: 34° 16′ 33.55» E and Alt: 1.020 m) in Konya province, Türkiye during the non-breeding season (February) in 2024. A total of 80 clinically healthy adult Merino ewes, aged 2–5 years and weighing between 50–60 kg, were included in the study. They were at 60–75 days postpartum. The ewes were allowed to graze on pasture for 12 h each day and were not provided with any compound feed. They had ad libitum access to water. No nutritional flushing was administered to the animals before mating. Lambs were fully weaned on day 0 before estrus stimulation.

### Synchronization protocol and treatment groups

Eighty ewes were randomly assigned to one of two groups: control and treatment. In treatment (Stop Lactin) group (n = 40), ewes were treated with Stop Lactin® (Raizup, Nutrinova, France) once, 60 ml on day 0 orally by syringe. The control group (Control) (n = 40), ewes were not treated with any anti-galactagogue feed supplements. On day 0, a vaginal sponge containing 60 mg of medroxyprogesterone acetate (Esponjavet®, Hipra, Spain) was inserted for seven days. On day 7, all ewes received an intramuscular injection of 500 IU PMSG (Oviser®, Hipra, Spain).

### Estrus detection

A teaser ram was used in 1-h sessions twice daily for 36 h to detect estrus after the sponges’ removal. Ewes identified as being in estrus were hand-mated with one of the proven fertile Merino rams, maintaining a ewe-to-ram ratio of 7:1.

### Blood collection and hormonal assessment

Blood samples were collected via the jugular vein using non-heparinized tubes on day 0 and day 7 from all ewes. The samples were subsequently centrifuged at 4.000 rpm for 10 min, and the obtained serum was stored at − 18 °C pending analysis. Determination of serum prolactin concentrations was performed by ELISA with a sensitivity of ≤ 0.221 ng/ml for samples between 0.25 and 70 ng/ml (Sheep Prolactin Sunred 201–07–0055).

### Ultrasonography examination

Pregnancy was determined transabdominally on day 50 post-mating using a real-time B-mode ultrasonography device with a convex probe (Hitachi EUB-405, 3.5 MHz, Japan). The litter size was determined at parturition.

### Determination of reproductive performance

Estrus rate, pregnancy rate, lambing rate, multiple birth rate and litter size were calculated as reproductive parameters as follows;

$$Estrus rate=\frac{the\;number\;of\;ewes\;showing\;estrus\;behaviours}{the\;number\;of\;ewes\;receiving\;intravaginal\;sponge}x100$$

$$Conception rate=\frac{the\;number\;of\;pregnant\;ewes}{the\;number\;of\;mated\;ewes}x100$$

$$Pregnancy rate=\frac{the\;number\;of\;pregnant\;ewes}{the\;number\;of\;ewes\;receiving\;intravaginal\;sponge}x100$$

$$\mathrm{Late}\;\mathrm{embryonic}-\mathrm{early}\;\mathrm{fetal}\;\mathrm{mortality}\;\mathrm{rate}=\frac{\mathrm{number}\;\mathrm{of}\;\mathrm{detected}\;\mathrm{death}\;\mathrm{embryo}-\mathrm{fetus}}{the\mathit\;number\mathit\;of\mathit\;pregnant\mathit\;ewes}\times100$$

$$Lambing rate=\frac{the\;number\;of\;lambing\;ewes}{the\;number\;of\;pregnant\;ewes}x100$$

$$Twin rate=\frac{the\;number\;of\;twin\;lambing\;ewes}{the\;number\;of\;pregnant\;ewes\;in\;each\;group}x100$$

$$Litter size=\frac{the\;number\;of\;total\;lambs}{the\;number\;of\;lambing\;ewes}x100$$

### Statistical analysis

Statistical analyses were performed using SAS (Version 8.0). Reproductive parameters were assessed using the Chi-squared test, Fisher’s exact test, and PROC GENMOD procedure. Normality of serum prolactin concentrations within each group was evaluated using the Shapiro–Wilk test. Differences in serum prolactin levels across different days were analyzed using the PROC MIXED procedure. Results were reported as percentages or means ± standard error of the mean (± SEM). Statistical significance was defined as p < 0.05, and tendency was considered for 0.05 < p ≤ 0.10.

## Results

After grouping, one ewe from the treatment (Stop Lactin) group died and was subsequently excluded from the study.

**Fig. 1:**
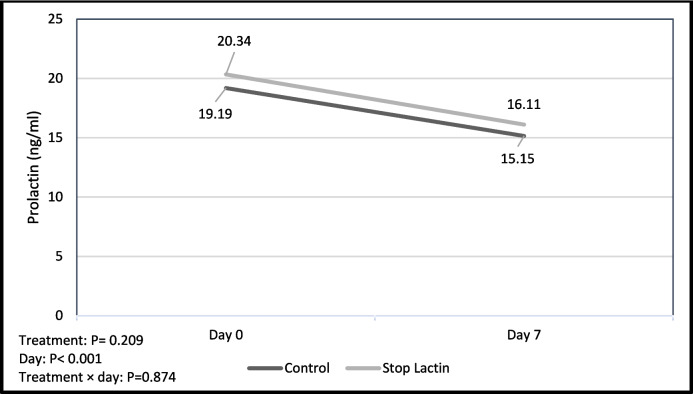
Serum prolactin concentrations in ewes (ng/ml)

Results for estrus rate, conception rate, pregnancy rate, late embryonic-early fetal mortality rate, lambing rate, twin rate, number of lambs and litter size are given in Table 1. Serum prolactin concentrations summarized in Table 2 and Fig. 1 by group. The treatment effect (P_treatment_ = 0.209) and the treatment × day interaction effect (P_treatment×day_ = 0.874) of the groups on serum prolactin level were found to be insignificant. However, the day interaction effect (P_day_ < 0.001) was found to be significant.

**Table 1:** Reproductive performance of ewes

Parameters	Control Group (n = 40) (X/n)	Stop Lactin Group (n = 39) (X/n)	*P*
Estrus rate (%)	97.5 (39/40)	82.1 (32/39)	0.050*
Conception rate (%)	87.2 (34/39)	78.1 (25/32)	0.316
Pregnancy rate (%)	85.0 (34/40)	64.1 (25/39)	0.037**
Late embryonic-early fetal mortality rate (%)	20.6 (7/34)	8.0 (2/25)	0.199
Lambing rate (%)	79.4 (27/34)	92 (23/25)	0.199
Twin rate (%)	29.6 (8/27)	13.0 (3/23)	0.168
Number of Lambs	27	23	
Single	19	20	
Twin	8 (16)	3 (6)	
Litter Size	**1.30 (35/27)**	**1.13 (26/23)**	**0.154**

^**^P > 0.05 is considered as non significant

^*^P 0.05 < p ≤ 0.10 considered as tendency

**Table 2:** Serum prolactin concentrations (ng/ml) on different days in ewes

Day	Control Group	Stop Lactin Group	*P*
Day 0	19.19 ± 0.75	20.34 ± 0.82	0.267
Day 7	15.15 ± 0.65	16.11 ± 0.65	0.354
Treatment 0.209			
Day < 0.001			
Treatment × day 0.874

## Discussion

Innovative, science-based methods are essential to ensure udder health and animal welfare during dry-off and a successful transition to the new lactation period (Bertulat et al. 2017). Stop Lactin® (Raizup, Nutrinova, France) is a herbal mixture prepared based on the lactation suppressant properties of sage, the inhibition of parsley leaves on the prolactin hormone, and the positive effects of echinacea on somatic cell numbers and immunoglobulin in the serum. In the product's prospectus, it is claimed that it reduces milk yield in cows, goats and ewes.

In parsley leaf oil, the main constituent is p-1,3,8-menthatriene, followed by β-phellandrene, myristicin, and β-myrcene. In parsley root oil, the predominant component is apiole, with significant amounts of myristicin and terpinolene as well (Kumar et al. 2015). Phytochemical constituents and compounds have been isolated from seeds, roots, leaves, or petioles using bioassay-guided methods. These constituents include essential oils, flavonoids, carbohydrates, furocoumarins, and other miscellaneous compounds (Agyare et al. 2017). Parsley enhances hormonal balance in women, increases their libido and estrogen secretion (Ajmera et al. 2019). Myristicin and apiole found in parsley have properties that can increase estrogen production, potentially affecting the uterus and stimulating the menstrual cycle (Ajmera et al. 2019; Awe and Banjoko 2013). However, consumption in large quantities can exert an uterotonic effect, making its use contraindicated during pregnancy. Ingesting more than 10 drops of the oil per day may lead to abortion in rats (Awe and Banjoko 2013). Parsley has been utilized to induce menstrual flow and address menstrual disorders (Ajmera et al. 2019; Bisset and Wichtl 1994). Moreover, parsley tea reduces menstrual issues and discomfort (Ajmera et al. 2019). However, experimental research has been scarce to support its folkloric use (Akpaso et al. 2023). Parsley is also commonly used by lactating mothers as a galactofuge to reduce excessive milk production (Awe and Banjoko 2013). Parsley is believed to lower prolactin levels and could decrease milk supply when consumed as food (Eglash 2014; Schaefer et al. 2007).

Sage is believed to suppress lactation and has been used to assist with weaning or to reduce an excessive milk supply (Amir et al. 2011; Eglash 2014; Scott and Jacobson 2005; Yarnell 1997). Sage tea or extract made from the leaves is commonly recommended, although there is a lack of studies on the use of sage for hypergalactia (Drugs and Lactation Database (LactMed®) [Internet] 2006). While one source on indigenous East Indian plants mentioned sage as useful for drying up milk during weaning, another did not reference such an action (Yarnell 1997). In an Ethnoveterinary medicines research in British Columbia, Canada, it was reported that sage was used to dried off goats. The method is as follows, a mixture of 1 teaspoon of dried sage in water is applied to the udder. Alternatively, the same amount of dried sage can be crumbled onto grain with molasses to make it more palatable for the goats (Lans et al. 2007). No scientific studies were found that assess the impact of sage on milk production (Drugs and Lactation Database (LactMed®) [Internet] 2006). Sage has gained a reputation as an antilactagogue, but this assertion is largely unsubstantiated by readily available findings in medical literature (Yarnell 1997). Moreover sage is specifically contraindicated during pregnancy due to its potential to induce abortion (Lawrence and Lawrence 2022). However, the mechanisms of action of the aforementioned plants require experimental validation for confirmation (Mohanty et al. 2014).

It is well known that prolactin is a lactogenic and mammogenic hormone secreted by the anterior pituitary. These effects were first discovered in 1929, when pituitary extract injected into rabbits stimulated udder growth and lactation (Riddle et al. 1933). Prolactin has been found to be crucial for initiating lactation during the periparturient period in various species, including cattle. In cattle, lactogenesis remains the only well-established function of prolactin to date (Tucker 2000). For instance, there was a rise in prolactin secretion several hours before parturition (Ingalls et al. 1973). Blocking this surge with bromocriptine significantly decreased subsequent milk yield, but administering exogenous prolactin reversed the bromocriptine effect (Akers et al. 1981). Prolactin release reaches peak levels with birth and is induced by nursing or milking. Prolactin release is under the influence of many factors. Especially in cows that are housed with their calves during the postpartum period, prolactin levels are higher than in cows whose calves are kept separately (Akers and Lefcourt 1982). So it may be summarized that prolactin's crucial role in triggering lactation during the periparturient phase has been revealed across various species, notably in cattle.

There are very few studies investigating prolactin and its effects on reproductive efficiency in ewes. In an early study, it was found that bromocriptine did not influence milk yield in goats (Hart 1973). However, a later study showed a 21% decrease in milk production after 8 days of bromocriptine treatment (Knight et al. 1990). Knight (1993) treated for six days using bromocriptine either alone or in conjunction with ovine prolactin. The results showed that milk production decreased by 13% when bromocriptine was administered alone, but only by 2% when bromocriptine was administered together with prolactin. Saat et al. (2024) reported that in their study daily oral administration of cabergoline did not significantly reduce prolactin levels in lactating Kıvırcık ewes. Ascari et al. (2013) reported that prolactin concentrations were not different between the groups early weaned, restricted and unrestricted Santa Ines ewes with their lambs. In this study, we investigated for the first time the effectiveness of a herbal mixture containing sage, parsley, and echinacea on serum prolactin levels and fertility in ewes during the non-breeding season. In the study presented, no significant differences were observed between the groups in serum prolactin concentrations on day 0 and day 7. Serum prolactin concentration in the Stop Lactin Group was expected to decrease on the day 7 compared to the control group due to sage and parsley mixture, but this did not occur (P_Treatment×day_ > 0.05). As can be seen from Fig. 1, Stop lactin treatment has no effect on serum prolactin concentration between groups. However, there is a decrease in serum prolactin concentrations from day 0 to day 7 in both groups. This decrease is related to the weaning of lambs in both groups at the beginning of the experiment. Stop lactin is recommended for dried off high-yielding cows and is used in dairy farms. However, the physiological mechanism by which parsley and sage reduce/cut milk yield is not reported in the literature.

In the presented study, it was hypothesized that parsley and sage would reduce milk yield and prolactin concentration and, as a result, increase fertility. Parsley and sage also have estrogenic effects. Bruce and Ramírez (2008) found that estrogen's inhibitory effect on lactation in rats occurs at the mammary gland level rather than at the pituitary gland level. They speculated that estrogen might modify or hinder the effects of oxytocin or glucocorticoids on mammary function. In fact, further studies may require on this subject. In ewes elevated prolactin levels during the postpartum period may inhibit gonadotropins, potentially preventing pregnancy (Mandiki et al. 1990). It has also been reported that during postpartum anestrus in Prealpes du Sud ewes, there is a negative correlation between prolactin concentrations and LH-induced ovulation mechanisms (Kann and Martinet 1975). Peclaris (1988) also showed that increased prolactin levels could inhibit ovarian activity during lactation in Karagounico ewes. These researchers demonstrated that inhibiting prolactin secretion with bromocriptine resulted in an earlier onset of ovarian activity. On the other hand, there are also studies reporting the opposite effects. In a recent study, Saat et al. (2024) reported that daily oral administration of cabergoline did not improve estrus response and pregnancy rates in lactating Kıvırcık ewes. Previously, Ascari et al. (2013) determined that the rate of first estrus until the 60th postpartum day was 54.5%, 50% and 63.6% in early weaned, restricted and unrestricted Santa Ines ewes with their lambs, respectively (p˃0.05). Akpaso et al. (2023) demonstrated a notable rise in estrogen levels in a study where rats received oral parsley leaf extract for 28 days. Conception rate and pregnancy rate, which are reproductive parameters, are similar parameters. When the conception rate is evaluated alone in the presented study, no statistical differences between the groups were obtain. However, the pregnancy rate is statistically lower in the stop lactin group. We think that this situation is related to the fact that the estrus rate tends to be low in the stop lactin group, and that it affects the pregnancy rate because the ewes in the Stop lactin group show less estrus. Parsley and sage are also known to have a phytoestrogenic effects. However, the mechanism that increases estradiol sensitivity is unknown (McNeilly 2001). Earlier reports (Moss et al. 1980; Restall and Starr 1977; Wright et al. 1981) have indicated an increased inhibitory effect of estradiol on LH release in postpartum ewes. However, conflicting results have been reported in other studies. Nevertheless, it has been noted that estradiol can stimulate hypothalamic activity, thereby enhancing pulsatile LH secretion in postpartum ewes (Wright et al. 1983). Mandinki et al. (1990) reported that the higher plasma estrogen concentrations in dry ewes compared to suckling ewes in early postpartum are the primary cause of the delayed return to estrus. In the present study the possible phytoestrogenic effect of Stop Lactin may have enhace the inhibitory effect of estradiol on LH secretion in lactating ewes. As a result, the estrus rate was low and therefore the probability of pregnancy is expected to be decreased.

## Conclusion

The results of this study, which for the first time investigated the effects of anti-galactagogue (sage, parsley extract) and anti-inflammatory (echinacea extract) feed supplements in lactating Merino ewes, showed no reduction in prolactin concentration and no improvement in reproductive performance parameters. We think that further studies to evaluate detailed parameters are needed to reveal the anti-galactagogue effect of parsley and sage and their effect on fertility.

## Data Availability

Further information on the data and methodologies will be made available by the author for correspondence, as requested.
